# Miocardiopericarditis en la enfermedad de still del adulto

**DOI:** 10.47487/apcyccv.v1i3.65

**Published:** 2020-09-30

**Authors:** Manuel Horna N, Rubén Romero, César Larrauri, Fernando Córdova, Jorge Martín Mispireta, Jorge Alarcón

**Affiliations:** 1 Servicio de Cardiología, Clínica Delgado - AUNA; Lima, Perú Servicio de Cardiología Clínica Delgado - AUNA Lima Perú; 2 Servicio de Reumatología, Clínica Delgado - AUNA; Lima, Perú Servicio de Reumatología Clínica Delgado - AUNA Lima Perú

**Keywords:** Enfermedad de Still del Adulto, Pericarditis, Miocarditis, Still´s Disease, Adult-Onset, Pericarditis, Myocarditis

## Abstract

El compromiso cardíaco en la enfermedad de Still del adulto (ESA) usualmente se manifiesta como enfermedad pericárdica, la cual, generalmente, tiene un curso benigno. La miocarditis es una complicación infrecuente con una prevalencia del 7%. El diagnóstico de ESA se basa en los *sets* de criterios de Yamaguchi o Fautrel. El tratamiento con corticosteroides y metrotexato es la primera y segunda línea terapéutica, respectivamente, que combinados son efectivos en 70% de los casos. Reportamos un caso de ESA de presentación inusual por el compromiso tanto miocárdico como pericárdico.

## Abstract (en)

Cardiac involvement in Adult-onset Still’s Disease (AOSD) usually manifests as a pericardial disease, that has a benign course. The myocarditis is a rare complication with 7% of prevalence. The diagnosis of AOSD is based in the Yamaguchi or Fautrel criteria. The treatment with steroids and methotrexate is the first and second therapeutic lines, respectively, the combination is effective in 70% of cases. We report a case of AOSD with unusual presentation due to myocardial and pericardial commitment.

La enfermedad de Still del adulto (ESA) es un raro desorden sistémico de tipo autoinmune ^1^; inicialmente fue descrita por George Still en 1897, como una artritis idiopática juvenil en niños ^2^. Posteriormente, en 1971, Eric Bywaters reporta casos de pacientes adultos cuyo curso clínico era semejante a la enfermedad de Still pediátrica ^3^. Se estima una incidencia de 0,16-0,4/100 000 habitantes; afecta a ambos sexos por igual y su aparición es más frecuente en los grupos etarios entre 15 a 25 y 36 a 46 años ^1^. Sus características principales son: fiebre alta, *rash* evanescente, dolor de garganta, poliartralgias o artritis, serositis, linfadenopatías, hepatoesplenomegalia, leucocitosis, elevación de neutrofilos, velocidad de eritrosedimentación elevada, alta ferritina sérica y enzimas hepáticas elevadas ^3^.

El diagnóstico de ESA es difícil de establecer por lo heterogéneo de la clínica y el amplio espectro de diagnóstico diferencial ^4^. El tratamiento es empírico e incluye antiinflamatorios no esteroideos, corticoesteroides, metotrexato por vía oral o parenteral y, para casos refractarios, anticuerpos monoclonales (agentes biológicos cuyo mecanismo es el bloqueo de citoquinas específicas) ^5^^,^^6^.

## Descripción del caso

**Figura 1:**
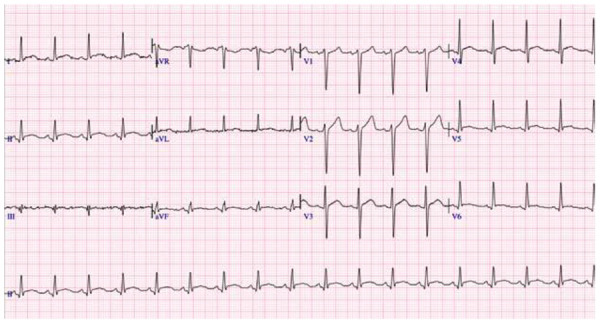
Electrocardiograma muestra taquicardia sinusal, desnivel del segmento PR y elevacion del punto J, y aplanamiento del ST en cara lateral baja.

**Figura 2:**
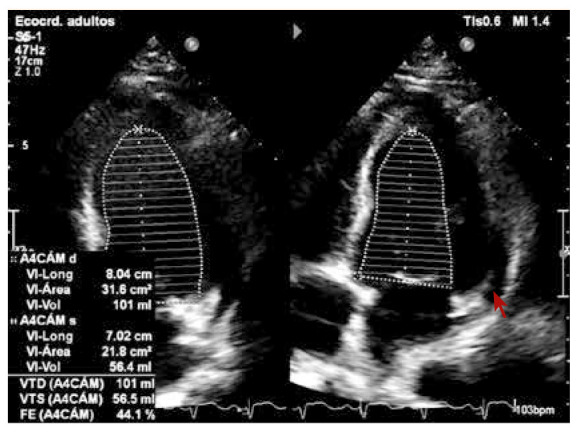
Vista de ecocardiograma de cuatro camaras que muestra FEVI 44% y efusion pericardica posterolateral leve.

Paciente varón de 27 años de edad, sin antecedentes cardiovasculares de importancia, procedente de Canadá, quien presentó episodio de faringoamigdalitis febríl 10 días antes de su viaje a Lima, Perú. Durante el vuelo de Canadá a Lima presenta dolor torácico opresivo intenso, por lo que es transferido a la emergencia de nuestra institución; al ingreso persistía el dolor torácico y refería disnea de reposo, fiebre y diarrea. A su ingreso los exámenes de laboratorio mostraron: leucocitosis con desviación izquierda asociada a linfopenia; hipoxemia y velocidad de sedimentación globular (VSG) elevada; el electrocardiograma (EKG) de ingreso mostró taquicardia sinusal con una frecuencia cardiaca en 106 lpm, con un intervalo QT en 480 ms, el segmento PR desnivelado y punto J elevado de manera difusa **(**Figura 1**)**. El estudio ecocardiográfico mostró fracción de eyección del ventrículo izquierdo (FEVI) en 44%, con hipocinesia global leve a moderada; patrón de llenado tipo normal; reflujo valvular mitral ligero y engrosamiento pericárdico a predominio pósterolateral con efusión pericárdica pósterolateral leve **(**Figura 2**)**. Los valores de troponina al ingreso y a las 48 h se encontraban dentro de la normalidad (0,013 y 0,06 ng/mL, respectivamente); asimismo, se realizó el dosaje de ProBNP con valores normales al ingreso y a los 12 días del internamiento (120 y 165 pg/mL respectivamente).

Una vez estabilizado el paciente y con el diagnóstico preliminar de miocarditis más pericarditis febríl, se consideró necesario descartar la etiología infecciosa o alguna enfermedad tropical por antecedente de estadías previas en zonas tropicales de Colombia y Panamá.

A los 12 días de hospitalizado continuaba febril con leucocitosis (> 20 000/mm^3^) y neutrofilia. La serología viral, los cultivos y el descarte de malaria fueron negativos. La ecocardiografía de control no mostró mejoría de la FEVI, manteniéndose alrededor de 45%. Al día 14 de hospitalización se objetivó *rash* maculopapular evanescente de tono rosado asalmonado ubicado en axilas y regiones laterales del tórax, asociado con poliartralgias y fiebre vespertina.

Todos los marcadores serológicos fueron negativos a pesar de elevarse mucho los reactantes de fase aguda (ej. ferritina sérica: 2000 ug/L). Fue así que, considerando los hallazgos clínicos y de laboratorio, en ausencia de otros diagnósticos, se realizó el diagnóstico de ESA, cumpliendo los criterios de Yamaguchi ^7^**(**Tabla 1**)**, tres criterios mayores: fiebre > 39 ºC por más de siete días, *rash* evanecente, y leucocitosis > 10 000 x mm^3^ y polimorfonucleares > 80 %; además, tres criterios menores: odinofagia, disfunción hepática (transaminasas elevadas) y anticuerpos antinucleares y factor reumatoideo negativos.

**Tabla 1:** Criterios de Yamaguchi

Criterios mayores	Criterios menores
Fiebre > 39 °C, con duracion > 7 dias	Dolor de garganta
Artralgias o artritis, con duracion > 15 dias	Linfadenopatias y/o esplenomegalia
Rash tipico evanescente	Pruebas hepaticas anormales
Leucocitosis > 10 000 x mm3 y con 80% PMN	ANA y FR negativos

Cinco o más criterios que incluyan dos o más criterios mayores

PMN: polimorfonucleares; ANA: anticuerpos antinuclerares; FR: factor reumatoideo

Adaptado de la referencia 14

**Figura 3:**
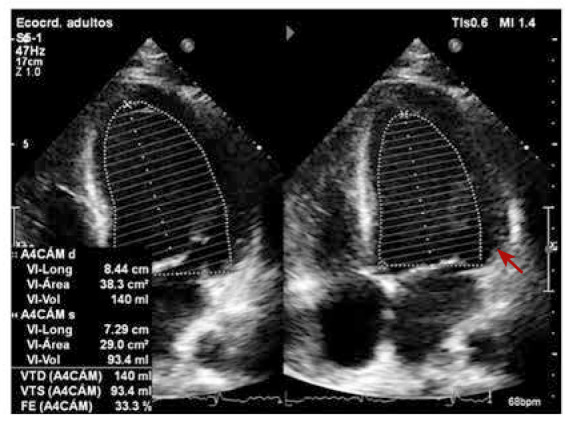
Vista de ecocardiograma de cuatro camaras que muestra FEVI 33%, sin efusion pericardica.

**Figura 4:**
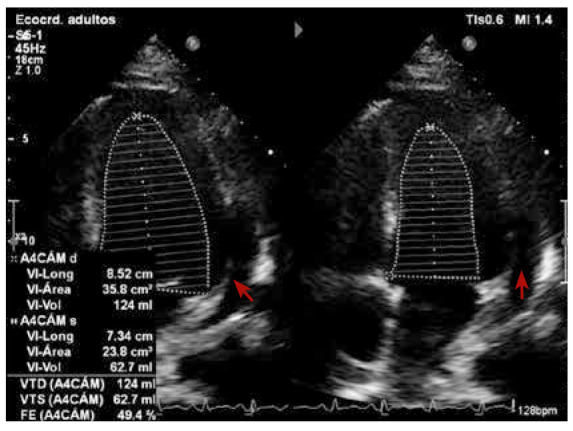
Vista de ecocardiograma de cuatro camaras que muestra FEVI 49% y efusion pericardica posterolateral leve, realizado al septimo dia de corticoterapia.

Al día 15 de la hospitalización se decide iniciar el tratamiento con metilprednisolona a 1 mg/kg/día EV, lográndose rápida resolución de la fiebre, del *rash* y de las poliartrálgias. Sin embargo, al quinto día de iniciado el tratamiento, el ecocardiograma de control mostró deterioro de la FEVI a 33%, sin evidencia de efusión pericárdica **(**Figura 3**)**. Al reajustarse la dosis a 1,5 mg/kg/día EV, se consiguió una evolución favorable, manteniéndose afebril, sin disnea ni dolor, disminución del valor de ferritina a 1190 ug/L y el ecocardiograma de control mostró una FEVI en 49% y efusion pericardica postero lateral leve **(**Figura 4**)**. Se realizó una resonancia cardiaca que no observó inflamación, se inició tratamiento con metotrexato y continuó en corticoterapia convirtiéndose dosis de metilprednisolona al equivalente de prednisona. El paciente retornó a su país y dos meses después reportó que la FEVI era de 58%, y que la ferritina y el resto de parámetros de laboratorio se encontraban en rangos normales.

## Discusión

Según los casos reportados en la literatura la ESA se manifiesta con pericarditis hasta en 20% de los pacientes, generalmente con evolución favorable; sin embargo, se han reportado algunos casos de taponamiento cardiaco ^8^; contrariamente, la miocarditis es infrecuente, con una prevalencia de 7% de los casos reportados. En el estudio de Gerfaud-Valentin *et al.*^9^, que compara sujetos con ESA sin miocarditis y ESA con miocarditis, la miocarditis ocurría temprano, estando presente al debut en 54% de los casos. Esta fue sintomática en la mayoría, con alteraciones inespecíficas en EKG y una FEVI ≤ 50%. La corticoterapia sola fue efectiva en 50% de los casos con miocarditis. Los pacientes con ESA complicada con miocarditis eran más jóvenes y en su mayoría varones, con relación al grupo sin miocarditis; en ellos la pericarditis fue más frecuente y mostraron leucocitos y niveles de ferritina más altos.

La edad de nuestro paciente es menor que el promedio de edad reportado por la literatura para pacientes con miocarditis asociada a ESA (27 vs. 32 años). El principal síntoma de nuestro paciente fue el dolor precordial, manifestado en el 58% de los pacientes de las series publicadas. La prevalencia de miocarditis en ESA es infrecuente, y se constituyó como uno de los diagnósticos ecocardiográficos resaltantes de nuestro paciente. La proporción de pacientes que cursan con falla cardíaca esta alrededor de 44%; el paciente reportado cursa con cuadro de disnea que levanta la sospecha clínica de falla cardíaca, la que es corroborada con los estudios de ecocardiografía, en los que se verifica una caída progresiva de la FEVI, desde el 45% hasta el 33%. Dentro de los marcadores inflamatorios, son la ferritina y la proteina C reactiva los que se incrementan, y nuestro paciente no fue la excepción, puesto que ambos valores se mantuvieron altos durante toda la hospitalización y solo mostraron un discreto descenso de 2000 a 1190 ug/L (ferritina).

A diferencia de las miocarditis por otra etiología, la FEVI se compromete con mayor frecuencia en los pacientes cuya causa de fondo es la ESA; asimismo, los marcadores cardioespecíficos (troponina) se elevan con mayor prevalencia en comparación a casos de miocarditis de otra etiología (94 vs. 34%) ^10^^)^ , en nuestro caso no encontramos dicha elevación probablemente porque solo se dosó la troponina al ingreso y a las 48 horas, por lo que no podemos afirmar o negar elevaciones posteriores.

En el estudio de resonancia magnética practicada no hubo hallazgos consistentes con inflamación miocárdica; la literatura reporta la ausencia de estos hallazgos inflamatorios hasta en el 36% de pacientes con miocarditis ^11^. Pudo haberse optado por pulsos de metilprednisolona a dosis entre 0,5 a 1 g por tres días, según consignan algunas series de casos. Se decidió, además, asociar metotrexato al alta; dicho esquema de tratamiento es compatible con las distintas referencias bibliográficas que reportan series de casos o casos aislados ^12^. Si hubiera sido refractario, la alternativa (tercera línea) era asociar al metotrexato con algún agente biológico de efecto inhibidor de la IL-1 o de IL-6 ^1^^,^^5^.

La miocarditis es una manifestación infrecuente de la ESA, lo cual genera retardo en el diagnóstico e inicio del tratamiento específico. A pesar de ello, el pronóstico de los pacientes con ESA y miocarditis asociada es alentador ya que la tasa de mortalidad reportada de la fase aguda alcanza el 4,2% que contrasta con el 21% de mortalidad reportada para cuadros de miocarditis en general ^13^. 
